# The identification and verification of hub genes associated with pulmonary arterial hypertension using weighted gene co-expression network analysis

**DOI:** 10.1186/s12890-022-02275-6

**Published:** 2022-12-13

**Authors:** Weibin Wu, Ai Chen, Siming Lin, Qiuran Wang, Guili Lian, Li Luo, Liangdi Xie

**Affiliations:** 1 Department of Geriatrics, The First Affiliated Hospital of Fujian Medical University, 20 Chazhong Road, Fuzhou, 350005 Fujian People’s Republic of China; 2 Fujian Hypertension Research Institute, The First Affiliated Hospital of Fujian Medical University, Fuzhou, People’s Republic of China; 3 Clinical Research Center for Geriatric Hypertension Disease of Fujian Province, The First Affiliated Hospital of Fujian Medical University, Fuzhou, People’s Republic of China; 4 Branch of National Clinical Research Center for Aging and Medicine, The First Affiliated Hospital of Fujian Medical University, Fuzhou, Fujian People’s Republic of China; 5 Department of Geriatrics, National Regional Medical Center, Binhai Campus of the First Affiliated Hospital, Fujian Medical University, Fuzhou, People’s Republic of China

**Keywords:** Hub genes, Pulmonary arterial hypertension, WGCNA, GSEA, Bioinformatics

## Abstract

**Background:**

Pulmonary arterial hypertension (PAH) is characterized by a progressive increase in pulmonary vascular resistance and pulmonary arterial pressure, with complex etiology, difficult treatment and poor prognosis. The objective of this study was to investigate the potential biomarkers for PAH based on bioinformatics analysis.

**Methods:**

The GSE117261 datasets were downloaded from the Gene Expression Omnibus database. Differentially expressed genes (DEGs) were identified by screening PAH patients and controls. Then the DEGs were analyzed using a Weighted Gene Co-expression Network Analysis (WGCNA) and the key modules were determined, and to further explore their potential biological functions via Gene Ontology analysis (GO), Kyoto Encyclopedia of Genes and Genomes Pathway analysis (KEGG), and Gene Set Enrichment Analysis (GSEA). Moreover, Protein–protein interaction (PPI) networks were constructed to identify hub gene candidates in the key modules. Finally, real-time quantitative polymerase chain reaction was supplied to detect the expressions of hub genes in human pulmonary arterial smooth cells treated with cobalt chloride (COCl_2_) which was used to mimic hypoxia.

**Results:**

There were 2299 DEGs identified. WGCNA indicated that yellow module was the key one correlated with PAH. GO and KEGG analysis demonstrated that genes in the yellow module were mainly enriched in ‘Pathways in cancer’. GSEA revealed that ‘HALLMARK_MYC_TARGETS_V1’ was remarkably enriched in PAH. Based on the PPI network, vascular endothelial growth factor A, proto-oncogene receptor tyrosine kinase (*KIT*), PNN interacting serine and arginine rich protein (*PNISR*) and heterogeneous nuclear ribonucleoprotein H1 (*HNRNPH1*) were identified as the hub genes. Additionally, the PCR indicated that the elevated expressions of *PNISR* and *HNRNPH1* were in line with the bioinformatics analysis. ROC analysis determined that *PNISR* and *HNRNPH1* may be potential biomarkers to provide better diagnosis of PAH.

**Conclusion:**

*PNISR* and *HNRNPH1* were potential biomarkers to diagnosis PAH. In summary, the identified DEGs, modules, pathways, and hub genes provide clues and shed light on the potential molecular mechanisms of PAH.

**Supplementary Information:**

The online version contains supplementary material available at 10.1186/s12890-022-02275-6.

## Introduction

Pulmonary arterial hypertension (PAH) is a kind of severe cardiovascular disease, characterized by the progressive increase in pulmonary vascular resistance and pulmonary arterial pressure, with complex etiology and poor prognosis, finally leading to right heart failure and death [1]. PAH is the term used to describe the finding of a mPAP ≥ 25 mmHg regardless of the underlying cause [2]. At present, the treatment strategies of PAH mainly include supportive therapy such as diuretics and oxygen therapy, medication therapies and surgeries such as heart and lung transplantation [3]. Regrettably, there is still no effective treatment for PAH, and the mortality remains high [4]. In recent years, studies of gene expression chips have been widely used to find potential biomarkers in complex diseases in order to further explore underlying pathogenesis and potential therapeutic targets [5].

A weighted gene co-expression network analysis (WGCNA) is used to identify correlations between genes and microarray samples [6] and the association of genes and clinical traits could recognize by clustering genes with similar expression profiles. Therefore, diseases-related hub genes could be discovered via WGCNA in cardiovascular diseases [7] and cancers [8].

In this study, the gene expression profiles of GSE117261 from 32 PAH patients and 25 controls were re-analyzed. The “limma” package in R software was used to obtain the DEGs. WGCNA was conducted to build a gene co-expression network. GO and KEGG enrichment analysis were performed in the key module and hub genes in the key module were determined and validated by Real-time quantitative polymerase chain reaction (RT-qPCR).

## Materials and methods

### Data Sources

**Fig. 1:**
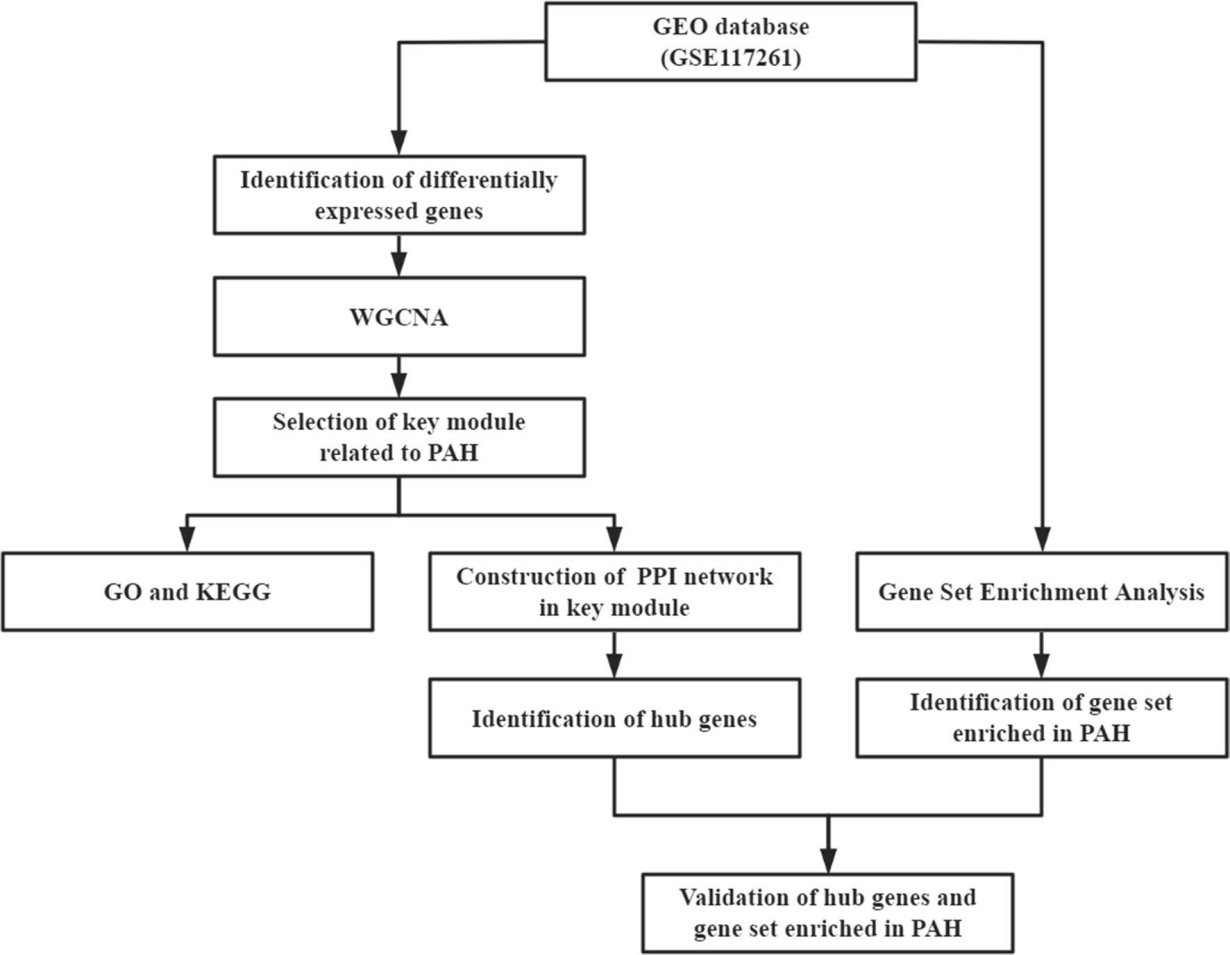
Workflow chart of the present study

Microarray expression data (GSE117261) [9, 10] was downloaded from the Gene Expression Omnibus (GEO) database (https://www.ncbi.nlm.nih.gov/geo/), representing a GPL6244 Affymetrix Human Gene 1.0 ST Array. GSE117261 includes 57 lung tissue specimens from 32 PAH patients and 25 normal controls. A workflow of the present study is presented in Fig. 1.

### Identification of differentially expressed genes

Data quality checking and normalization with log transformation were first performed to eliminate any batches. The “limma” package [11] in R software was used to screen DEGs between the PAH and control group. An adjusted *P*-value < 0.05 was set as the threshold criterion for statistical significance. The volcano map of DEGs was plotted in R software. The Heatmap package in R software was utilized to visualize the top 50 DEGs.

### Weighted gene co-expression network analysis

WGCNA [12] was conducted to build the co-expression network in DEGs based on the scale-free topology criteria. First, all DEGs were analyzed using the WGCNA package in R software, and the soft thresholding power was set. Next, the weighted co-expression network was constructed, and DEGs were clustered into several modules with different color labels. The correlation between each module and PAH or controls was then explored. The module most correlated with PAH was regarded as a key module for further enrichment analysis.

### Functional enrichment analysis of genes in the key module

Key module indicating the deep correlations with PAH were taken for further analysis. Base on the “org.Hs.eg.db” package and “cluster profiler” R package were installed in R software, GO and KEGG enrichment analysis was used to examine the gene complement of the key module [13–18]. The GO analysis annotates gene function contains: biological process (BP), cellular component (CC), and molecular function (MF). A *P*-value < 0.05 was considered statistically significant. Then bubble charts were used to display the top 10 enriched items from each GO category and from KEGG.

### Gene set enrichment analysis

Gene set enrichment analysis (GSEA) [19] was performed using the hallmark gene sets (h.all.v7.0.symbols), which were obtained from the Molecular Signatures Database (https://www.gsea-msigdb.orggsea/msigdb/index.jsp), and the analysis was used to detect whether the relevant biological pathways is statistically significant in PAH patients and controls. Transcriptome data were imported into the GSEA software v4.0.3. The cutoff point of significance was normalized enrichment score |NES|> 1, *P*-value < 0.05, false discovery rate (FDR) q-value < 0.25 for GSEA.

### Protein–protein interaction network and hub gene identification

The Search Tool for the Retrieval of Interacting Genes/Proteins (STRING, http://string-db.org/) was used to construct the protein–protein interaction (PPI) network of the key modules [20]. The PPI network was then visualized by Cytoscape software [21]. The Molecular Complex Detection (MCODE) [22] was used to screen the significant modules of the PPI network and then Cytohubba [23]was used to determine the hub genes.

### Isolation and culture of human PASMCs

Human samples were collected with a protocol approved by the Branch for Medical Research and Clinical Technology Application, Ethics Committee of the First Affiliated Hospital of Fujian Medical University (Approval No. MRCTA, ECFAH of FMU[2021]483). The clinical samples of human pulmonary arterioles were obtained from 10 male patients (60 ± 11 years old) who underwent lobectomy for lung cancer in the Department of Thoracic Surgery, The First Affiliated Hospital of Fujian Medical University, Fujian Province. The normal pulmonary arterioles of patients undergoing clinical lobectomy were taken, and the blood vessels were repeatedly cut with sterile ophthalmic scissors, and then placed in DMEM/F12 with 20% FBS at 37 °C and incubated a moist atmosphere of 5% CO2 at 37 ˚C. After 2 weeks, the well-grown cells show a typical "peak-valley" distribution. The cells were then passaged with 0.25% trypsin. When the cells were 70–80% confluent, the medium was replaced with DMEM and starved for 24 h. Then cells were treated with cobalt chloride (COCl_2_) at a final concentration of 100 μmol/L for 24 h to mimic hypoxia.

### RNA extraction and quantitative RT-PCR

Total RNA from PASMCs was extracted using the FastPure Cell/Tissue Total RNA Isolation *KIT* V2(Vazyme, Nanjing, China) according to the manufacturer’s instructions. The cDNA synthesis was conducted using Hifair III 1st Strand cDNA Synthesis SuperMix (Yeasen Biotech, Shanghai, China). Using β-actin as a reference, we performed quantitative RT-PCR with Hieff qPCR SYBR Green Master Mix (Yeasen Biotech, Shanghai, China) in the LightCycler® 96 System (Roche Diagnostics, Mannheim, Germany). The 2^−∆∆Ct^ method was applied to calculate the relative expression level of mRNA. The primer pairs used for amplification were as follows: *PNISR* F:5′-ATG TGG GAT CAA GGA GGA CAG-3′; R: 5′-CAG CCC AAT CAA TCT GGC TT-3′; *HNRNPH1* F:5′-GGA GGC TAT GGA GGC TAT GAT-3′; R:5′-CCT GTT GTG CTC TGG AAA GTAG-3′; GAPDH F:5′-GGT GTG AAC CAT GAG AAG TAT GA-3′; R:5′GAG TCC TTC CAC GAT ACC AAAG–3′.

### Statistical analysis

The GraphPad Prism 7 software (San Diego, CA, USA) were employed to analyze the data. Normal distribution measurement data were displayed as mean ± SD. The statistically significant differences between the PAH group and controls were performed by using Students t-test in GraphPad Prism 7. The construction of receiver operator characteristic (ROC) curve and the calculation of the area under the ROC curve (AUC) were finished in MedCalc Statistical Software version 14.8.1 (MedCalc Software Ltd, Ostend, Belgium). The statistical significance was set as *P* < 0.05.

## Results

### Identifications of DEGs

**Fig. 2:**
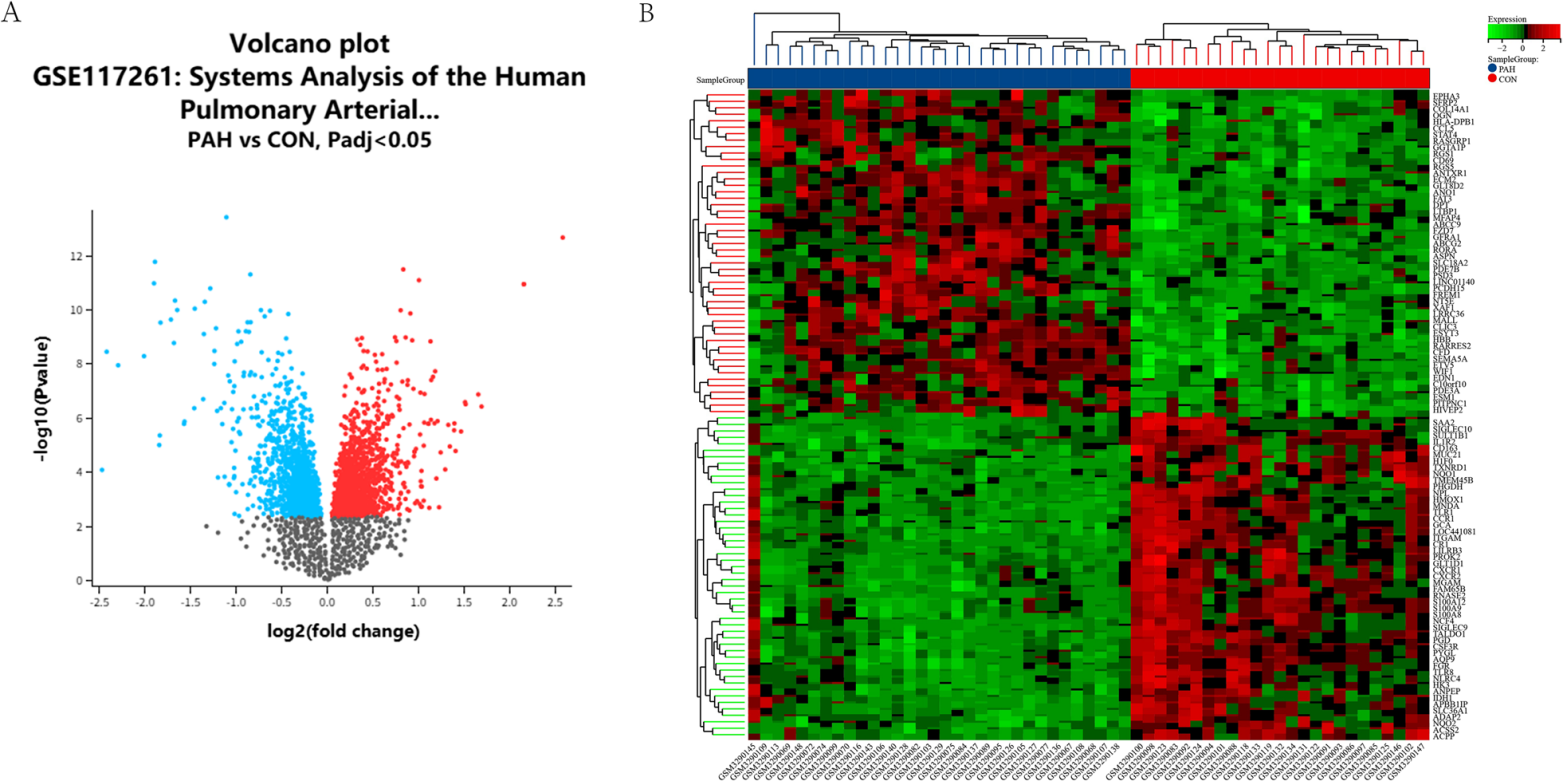
Expression profile of DEGs. **A** The volcano map of DEGs. Black dots represent genes that are not differentially expressed between PAH patients and control patients. Blue indicates down-regulated genes, and red indicates up-regulated genes. **B** Heatmap of top 50 DEGs. The red color indicates the higher gene expression value while the green color indicates the lower gene expression

A total of 2298 differentially expressed genes were identified, with an adjusted *P*-value < 0.05 between PAH patients and controls. Compared with the controls, there were 1140 genes were upregulated and 1158 genes were downregulated in PAH. The volcano map of DEGs is shown in Fig. 2A. The heatmap for the top 50 DEGs is displayed in Fig. 2B.

### WGCNA analysis

**Fig. 3:**
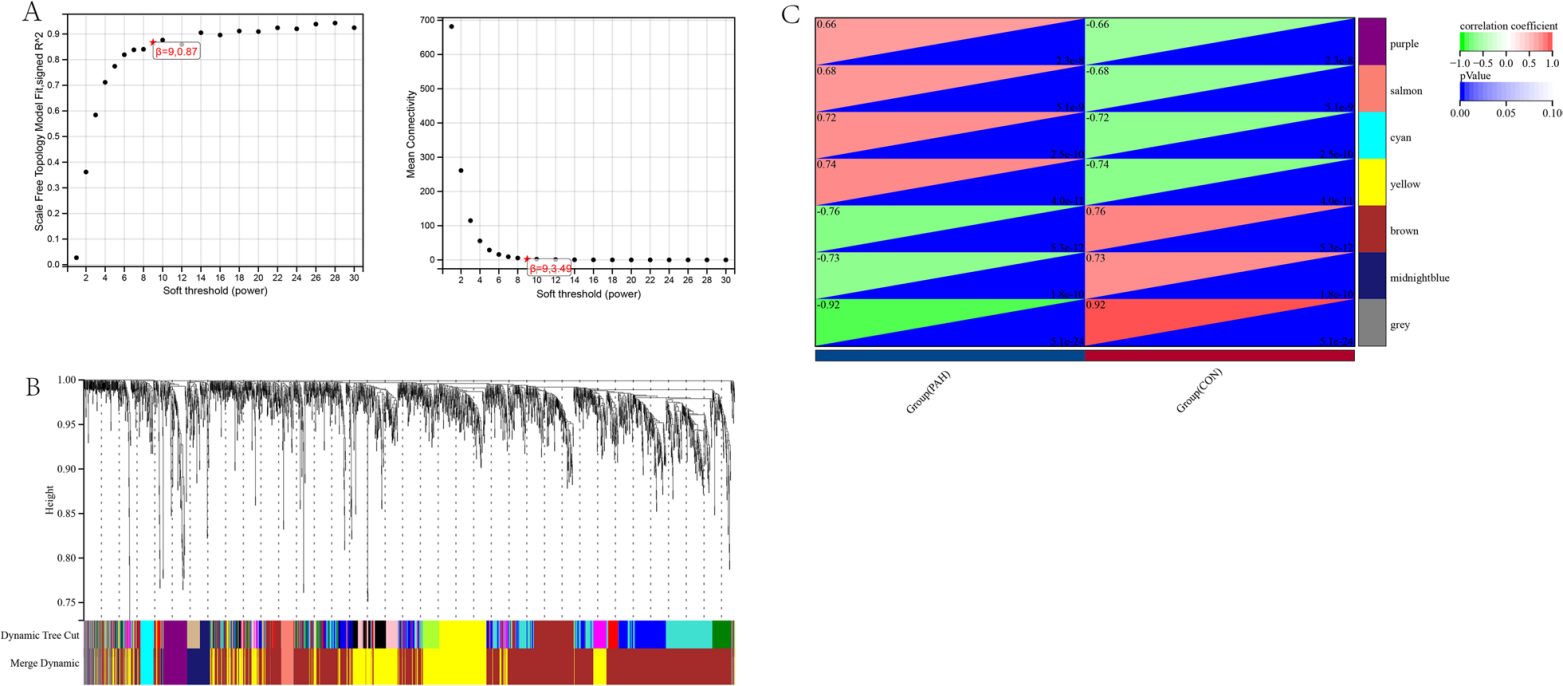
WGCNA of DEGs. **A** Estimation of the soft thresholding value for a scale-free coexpression network. **B** Cluster dendrogram of all DEGs. **C** Correlation between each module and patients or controls

The 2299 identified DEGs were further processed with the WGCNA package in R software, and a scale-free co-expression network (scale-free R^2^ > 0.8) was established using a soft thresholding power of 9. The soft thresholding power β was set at 24 in the subsequent analysis, because the scale independence reached 0.887 (Fig. 3A) and had a relatively good average connectivity. The DEGs were clustered into seven modules, purple, salmon, cyan, yellow, brown, midnight blue and grey, with a minimal module size > 30. The cluster dendrogram of the DEGs is shown in Fig. 3B. The correlation between each module and PAH was calculated and plotted (Fig. 3C). The results indicated that yellow (0.74, *P* < 0.0001) were the most positive modules related to PAH. Thus, the yellow module, including 597 DEGs, was considered as a key module correlated to PAH.

### Functional enrichment analysis

**Fig. 4:**
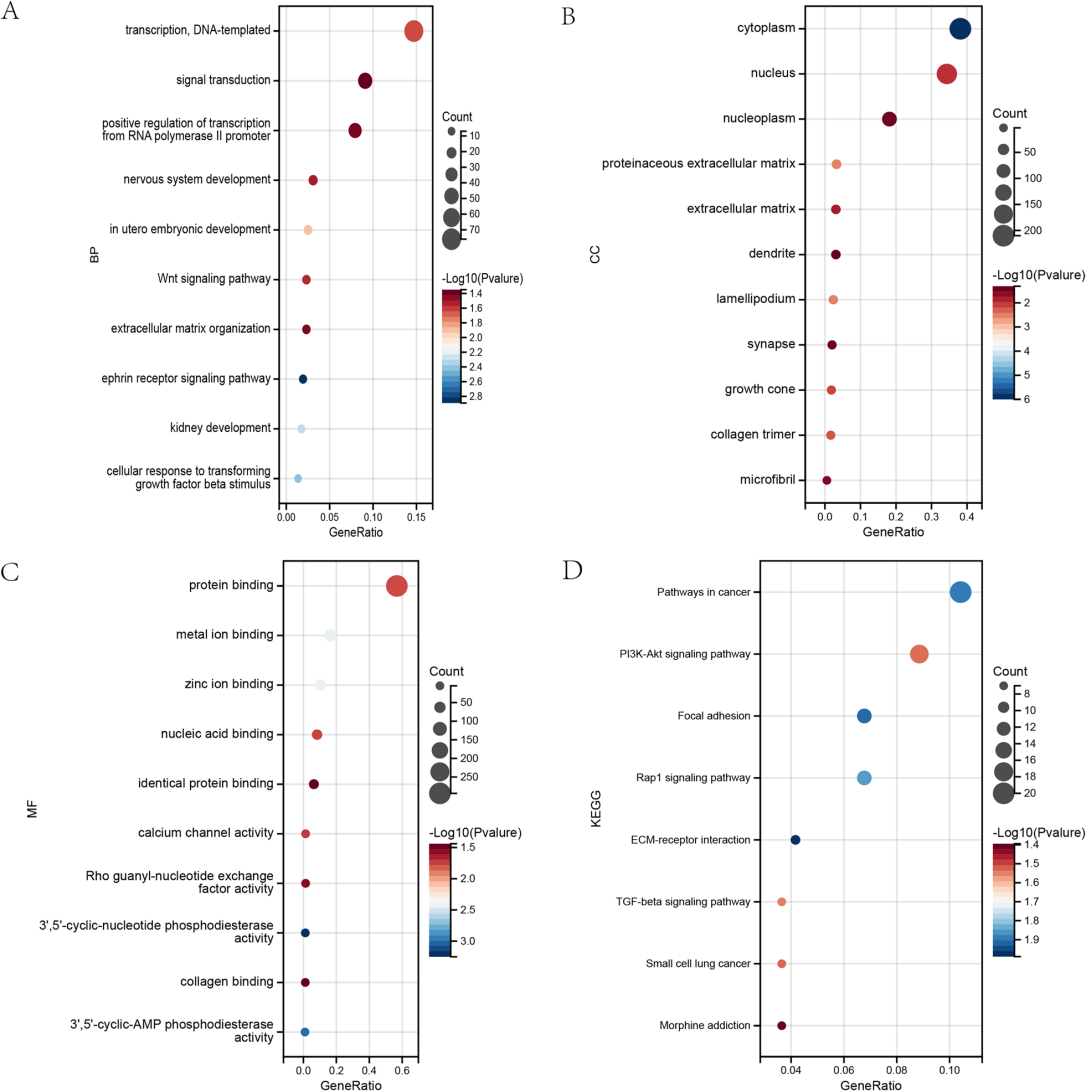
Gene ontology and KEGG enrichment analysis. **A** Biological process. **B** Cellular component. **C** Molecular function. **D** KEGG enrichment analysis [16–18]

The 597 DEGs in the yellow module were used for GO and KEGG analysis with the Metascape tool. GO enrichment reults indicated that the yellow module genes were enriched in ‘transcription, DNA-templated’, ‘signal transduction’ and ‘positive regulation of transcription from RNA polymerase II promoter’ in BP terms (Fig. 4A). In terms of CC, the terms ‘cytoplasm’, ‘proteinaceous extracellular matrix’ and ‘lamellipodium’ were significantly enriched (Fig. 4B). The MF terms of the DEGs were ‘protein binding’, ‘metal ion binding’ and ‘zinc ion binding’ (Fig. 4C). Moreover, KEGG analysis demonstrated that the yellow module was enriched in ‘Pathways in cancer’, ‘PI3K-Akt signaling pathway’, and ‘Focal adhesion’ (Fig. 4D).

### GSEA analysis

**Fig. 5:**
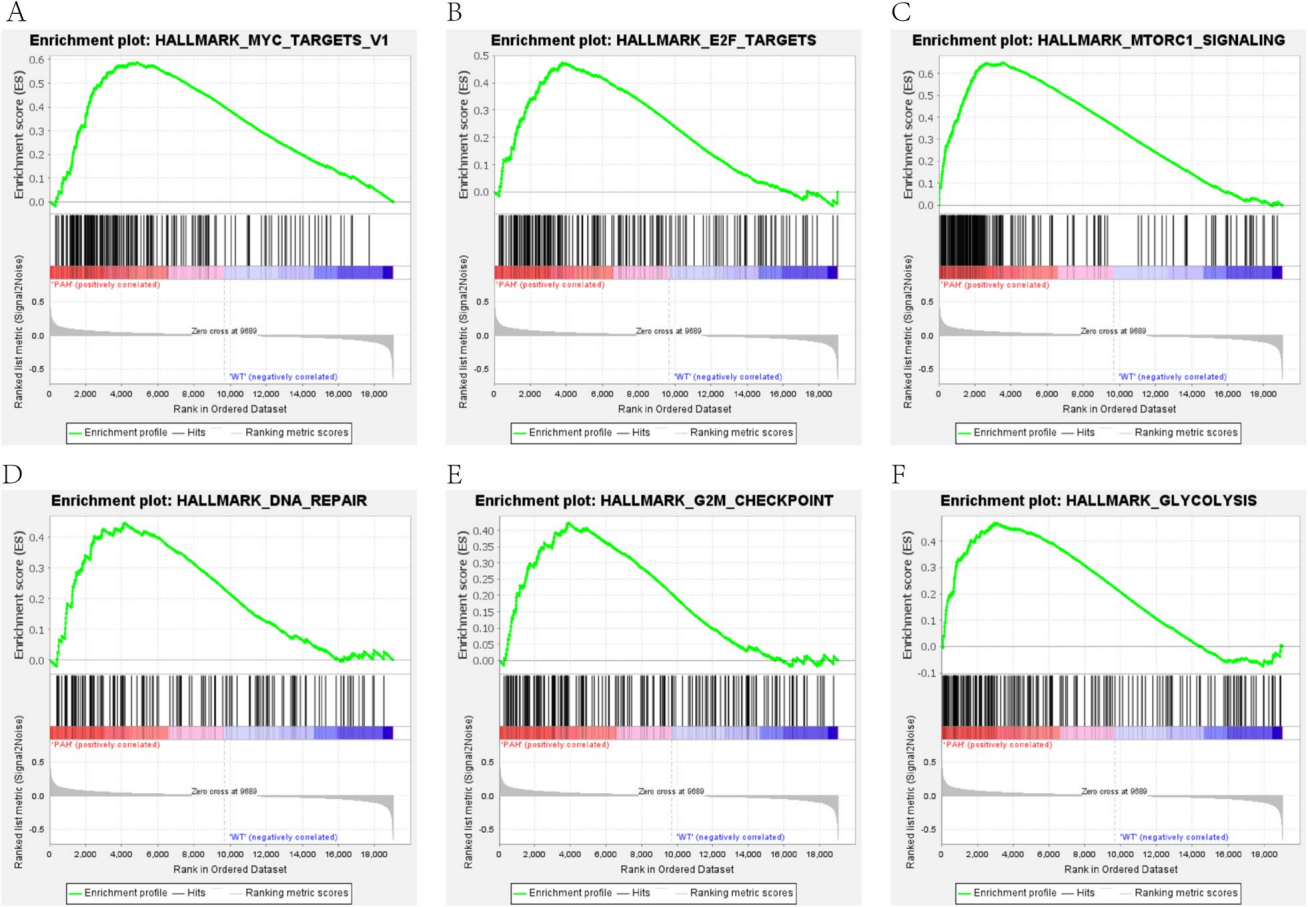
Gene set enrichment analysis. **A** Enrichment plot of ‘MYC_TARGETS_V1’ with enrichment score 0.59, FDR q-value 0.0. **B** Enrichment plot of ‘E2F_TARGETS’ with enrichment score 0.47, FDR q-value 0.0. **C** Enrichment plot of ‘MTORC1_SIGNALING’ with enrichment score 0.65, FDR q-value 0.0. **D** Enrichment plot of ‘DNA_REPAIR’ with enrichment score 0.45, FDR q-value 0.0. **E** Enrichment plot of ‘G2M_CHECKPOINT’ with enrichment score 0.42, FDR q-value 0.0. **F** Enrichment plot of ‘GLYCOLYSIS’ with enrichment score 0.47, FDR q-value 0.0

The distribution of the pathway gene sets on all gene expression data from the PAH patients and controls was explored using the GSEA software [19, 24]. The results showed that 27/50 gene sets were upregulated in the PAH patients, while 13 gene sets were significantly enriched with FDR < 25%. In the controls, 23/50 gene sets were upregulated, and 12 gene sets were highly enriched with FDR < 25%. The results of GSEA indicated that PAH patients enriched in ‘HALLMARK_MYC_TARGETS_V1’, ‘E2F_TARGETS’, ‘MTORC1_SIGNALING’, ‘DNA_REPAIR’, ‘G2M_CHECKPOINT’, and ‘GLYCOLYSIS’ pathway. The top six gene sets were shown in Fig. 5A–F.

### PPI network construction, modular analysis, and hub gene analysis

**Fig. 6:**
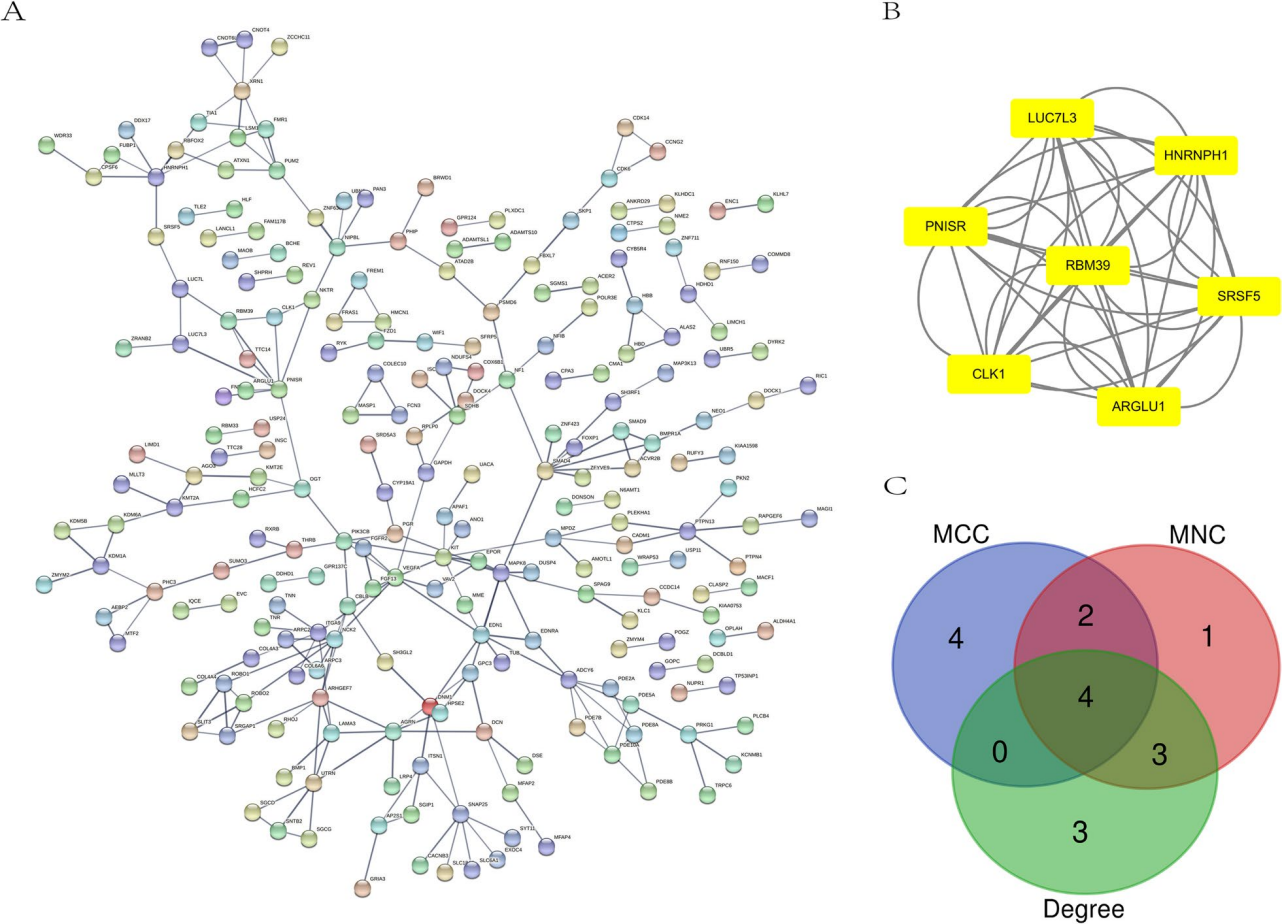
PPI network and hub gene. **A** PPI network. **B** The most significant modules. **C** The overlapped hub genes from different algorithms

The STRING database was used to construct the PPI network in order that we can explore the interaction of genes in the yellow module. Then, 0.7 was set as the threshold as the minimum required interaction score for constructing the STRING PPI network. As is shown in Fig. 6A, the PPI network comprised 230 nodes and 520 edges. Using the MCODE plug-in in Cytoscape, one module was determined. This Module (score = 7.0) included 7 nodes and 42 edges (Fig. 6B). The top ten hub genes obtained by MNC, MCC and Degree, in the cytohubba plug-in, are shown in Table 1. The overlapped hub genes among the three algorithms were verified by a Venn diagram (Fig. 6C), including *VEGFA*, *KIT*, *PNISR* and *HNRNPH1*.

**Table 1:** Top ten hub genes obtained by three algorithms of Cytohubba

MNC	MCC	Degree
*LUC7L3*	*VEGFA*	*GAPDH*
*HNRNPH1*	*GAPDH*	*VEGFA*
*SRSF5*	*KIT*	*KIT*
*PNISR*	*SMAD4*	*SMAD4*
*RBM39*	*PNISR*	*PNISR*
*CLK1*	*HNRNPH1*	*KDM6A*
*ARGLU1*	*SRSF5*	*NF1*
*VEGFA*	*LUC7L3*	*HNRNPH1*
*KIT*	*FGFR2*	*FGFR2*
*TIA1*	*MAPK8*	*XRN1*

### Validation of the hub genes

**Fig. 7:**
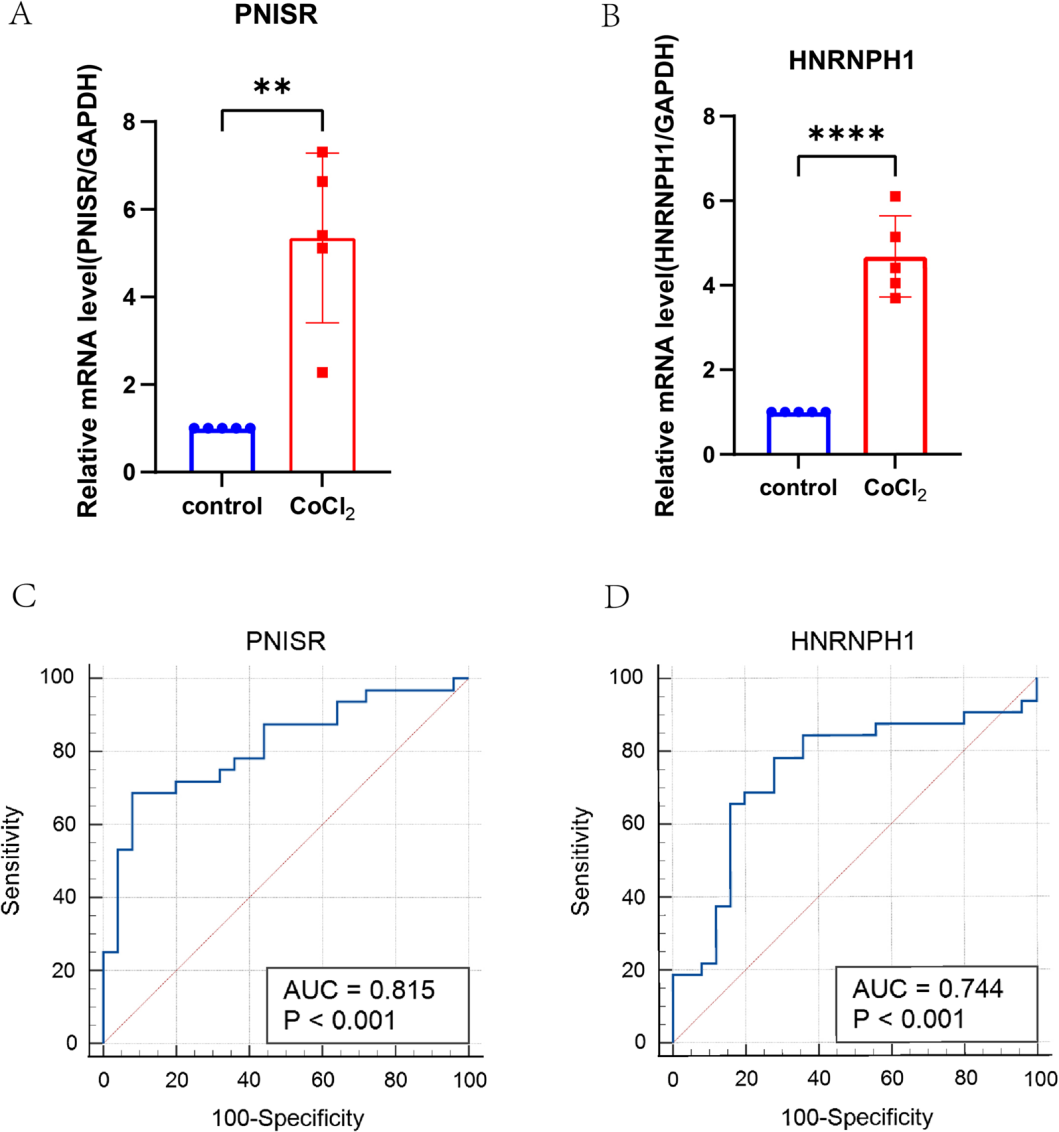
Validation of hub genes. **A** Relative mRNA level of *PNISR* in controls vs. PAH group. **B** Relative mRNA level of *HNRNPH1* in controls versus PAH group. (**P < 0.01, ****P < 0.0001). **C** ROC curve for *PNISR*. **D** ROC curve for *HNRNPH1*

Base on the quantitative RT-qPCR experiment, the transcriptional changes of overlapped hub genes *PNISR* and *HNRNPH1* were detected in the PASMCs from human by the method reported previously. The primary cultured PASMCs were divided into two groups: control group and CoCl_2_ treated group. The results indicated that the expression levels of *PNISR* and *HNRNPH1* were both upregulated in the CoCl_2_ treated in comparison with those in controls (Fig. 7A, B), which was in line with the bioinformatics analysis, and the similar results were observed in rats (Additional file 1: Fig S1).To evaluate the capability of *PNISR* and *HNRNPH1* to distinguish the CoCl_2_ treated from controls, ROC curves were performed. According to our data, the AUC of *PNISR* and *HNRNPH1* were 0.815 (95% *CI* 0.690–0.905; *P* < 0.0001) and 0.744 (95% CI 0.611–0.850; *P* = 0.0005), respectively, showing that the identified hub genes *PNISR* and *HNRNPH1* may be novel biomarkers of the PAH (Fig. 7C, D).

## Discussion

PAH remains one of the serious and fatal lung disease with high morbidity and mortality worldwide [25]. PAH is often characterized by elevated pulmonary artery pressure and pulmonary vascular resistance, which finally leads to progressive right heart failure and death [26]. PAH patients might suffer severe exertional dyspnea, angina, fatigue, weakness, angina, presyncope, and syncope [27]. Without prompt intervention, most people may die from various symptoms. Pulmonary hypertension remains a global health problem [28]. So, it is particularly important to find new etiologies, effective diagnostic markers and treatment strategies.

In this study, a WGCNA on mRNA expression profile GSE117261 downloaded from the GEO database was performed. Based on the WGCNA analysis, all 2299 DEGs obtained by the “limma” package in R software were clustered into 7 modules. Then, 597 DEGs in the yellow module were found to be the most positive genes related to PAH (correlation score = 0.74, *P* < 0.0001), which were mainly enriched in ‘cytoplasm’ and closely related to ‘transcription, DNA-templated’, ‘protein binding’ and ‘pathways in cancer’. GSEA showed that the gene set ‘HALLMARK_MYC_TARGETS_V1’ was obviously enriched in the PAH group. Four hub genes, vascular endothelial growth factor A (*VEGFA*), v-kit Hardy-Zuckerman 4 feline sarcoma viral oncogene homolog (*KIT)*, *PNN Interacting Serine and Arginine Rich Protein* (*PNISR)* and *Heterogeneous Nuclear Ribonucleoprotein H1* (*HNRNPH1*) were determined based on the PPI network. Meanwhile, upregulatd expressions of *PNISR* and *HNRNPH1* were validated by RT-qPCR.

Based on the GO analysis, the DEGs mainly consist of “Transcription, DNA-templated” in BP terms, which has been previously reported to be correlated to PAH [29]. The CC term “cytoplasm” and the MF term “protein binding” were both closely related to the transcription, which is an essential part of gene expression [30]. KEGG analysis revealed that the “Pathway in cancer” played a marked role in PAH, which was consistent with the previous demonstration demonstrated that the pathobiology of small vessels in severe PAH patients was quasi-neoplastic and cancer-like [31]. Meanwhile, the reliability of the DEGs obtained from the previous analysis was completely confirmed by results of the enrichment analysis.

By performing the GSEA analysis on the gene profile of GSE, many gene sets highly enriched in the PAH were found. The top six gene sets, including ‘HALLMARK_MYC_TARGETS_V1’, ‘E2F_TARGETS’, ‘MTORC1_SIGNALING’, ‘DNA_REPAIR’, ‘G2M_CHECKPOINT’, and ‘GLYCOLYSIS’ pathway, are cell cycle-related pathways, suggesting that genes involved in these pathways might contribute to cell proliferation. Among them, ‘HALLMARK_MYC_TARGETS_V1’ with an enrichment score of 0.59, was experimentally validated. This gene set involved 184 genes, including the well-known *proliferating cell nuclear antigen* (*PCNA*), *minichromosome maintenance protein* (*MCM*) and other cell proliferation markers [32]. Moreover, v-myc avian myelocytomatosis viral oncogene homolog (*MYC*) is a transcription factor known to regulate various human genes, promoting cell growth and proliferation [33], regulating apoptosis by altering pro- and anti-apoptotic members of the BCL-2 family and activating telomerase, controling the angiogenesis by regulating the expression levels of VEGF [34].

More and more studies showed that cell proliferation was one of the pathophysiological mechanisms of pulmonary arterial hypertension [35]. Inhibition of the proliferation of pulmonary artery smooth muscle cells can effectively ameliorate pulmonary arterial hypertension [36]. We are delighted to find that cell proliferation is enriched in PAH in accordance with the previous findings.

Based on previous studies, our work provides a new insight into the underlying pathogenesis of PAH. Dong H et al. identified *CSF3R, NT5E, ANGPT2, FGF7* and *CXCL9* as candidate biomarkers of PAH, and ruxolitinib might exert promising therapeutic action for PAH [37]. Qiu X et al. performed a WGCNA on GSE15197 and found that 11 real hub genes, including *EP300, MMP2, CDH2, CDK2, GNG10, ALB, SMC2, DHX15, CUL3, BTBD1,* and *LTN1*, were over-expressed in IPAH [38]. Contrastly, Liu J et al. suggested that 10 hub genes were determined via Cytohubba and a crucially ceRNA network was identified, including 14 LncRNAs, 2 miRNAs, and 3 mRNAs [39]. Interestingly, Li Q et al. confirmed that 9 hub genes related to PAH, particularly the *PLK4* and *SMC2* genes, providing a deeper understanding of physiopathologic of PAH [29]. Farha S et al. inferred that inhibition of *KIT* progenitor could improve remodeling and proliferation in PAH. Imatinib, a tyrosine kinase inhibitor that targets *c-KIT*, has been shown to be beneficial to PAH patients due to its inhibition of proliferation in hematopoietic progenitors and mast cells [40]. Liu J et al. found that *VEGF* initiated vascular remodeling resulting in PAH [41]. However, to our knowledge, research on the role of *PNISR* and *HNRNPH1* in PAH remains limited.

*PNISR* is a newly identified serine-arginine (SR) protein in human that co-purifies with pinin, which is rarely reported [42]. Sinclair PB et al. suggested that abnormal expressions of *GRIK2* and *PNISR* were associated with proliferation in some lymphoid leukemias [43]. *HNRNPH1*, a core member of the heterogeneous nuclear ribonucleo-proteins family, frequently upregulated in various cancer cells and contributed to tumorigenesis [44]. Elevated expression of *HNRNPH1* was found in acute myeloid leukemia (AML), and knockdown of *HNRNPH1* alleviated cell proliferation [45]. And the upregulated expressions of *PNISR* and *HNRNPH1* in PAH were found in our study. According to the ROC curve, the values of the AUC of *PNISR* and *HNRNPH1* were 0.815 and 0.744, respectively. The AUC result indicated that *PNISR* and *HNRNPH1* had a powerful ability to discriminate PAH from the controls. Together, *PNISR* and *HNRNPH1* were considered as candidate biomarkers of PAH. Nevertheless, further research are required to investigate the role of *PNISR* and *HNRNPH1* on the development of PAH, and the expansion of the sample size is needed to validate the efficacy of *PNISR* and *HNRNPH1* as biomarkers for PAH.

## Conclusions

Conclusively, *PNISR* and *HNRNPH1* were determined to be potential biomarkers in PAH. Our results indicate that *PNISR* and *HNRNPH1* participate in the development of PAH and serve as potential diagnosis and therapeutic targets for PAH.

## Supplementary Information

**Additional file 1.** Validation of hub genes in rat PASMCs.

## Data Availability

The dataset analyzed in this study can be derived from public repositories: GSE117261 dataset (https://www.ncbi.nlm.nih.gov/geo/query/acc.cgi?acc=gse117261).
