# Supplementary material for: The identification and verification of hub genes associated with pulmonary arterial hypertension using weighted gene co-expression network analysis
Source: BMC Pulm Med. 2022 Dec 13;22:474. doi: 10.1186/s12890-022-02275-6 (PMC9746192; doi:10.1186/s12890-022-02275-6)
Supplement: Supplementary file 1 — Additional file 1. Validation of hub genes in rat PASMCs. [file 12890_2022_2275_MOESM1_ESM.docx]

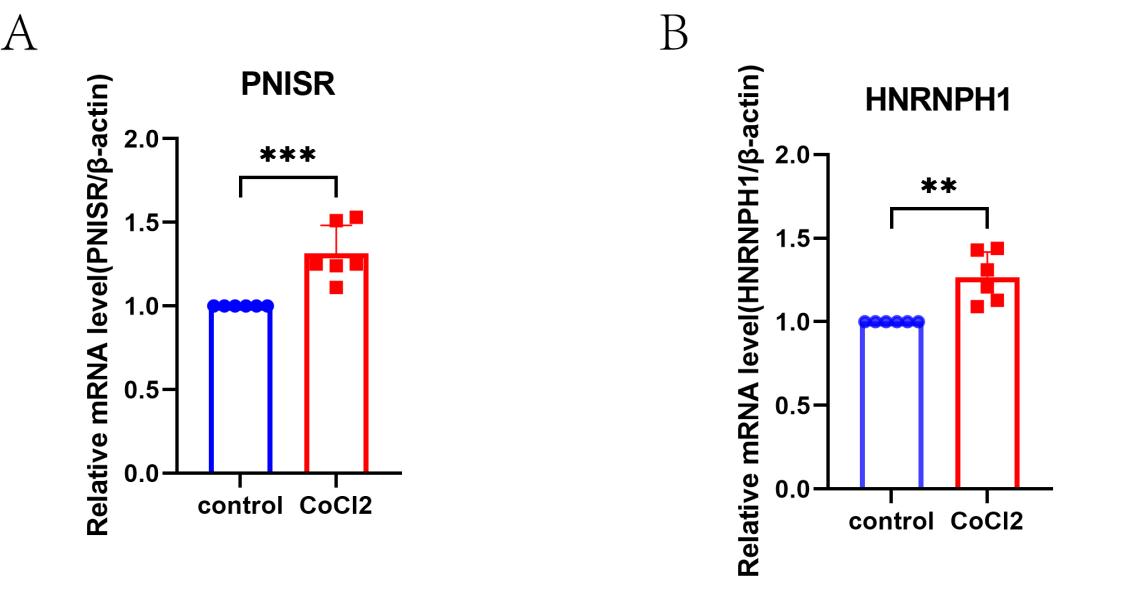


Supplementary Fig 1. Validation of hub genes in rat PASMCs. (A) Relative mRNA level of *PNISR* in controls vs. PAH group. (B) Relative mRNA level of *HNRNPH1* in controls vs. PAH group. (***P* <0.01, ****P* <0.001).
